# Case report: Multiple facial trichoepitheliomas caused by p.Val835SerfsTer52 variant of *CYLD* gene

**DOI:** 10.3389/fmed.2024.1458723

**Published:** 2024-09-30

**Authors:** Tatiana A. Gaydina, Olga I. Patsap, Anastasiia A. Buianova

**Affiliations:** ^1^Department of Dermatovenereology, Pirogov Russian National Research Medical University, Moscow, Russia; ^2^Federal Center of Brain Research and Neurotechnologies FMBA, Moscow, Russia; ^3^Center for Precision Genome Editing and Genetic Technologies for Biomedicine, Pirogov Russian National Research Medical University, Moscow, Russia

**Keywords:** multiple trichoepitheliomas, Brooke–Spiegler syndrome, CYLD, c.2501dupC, p.Val835SerfsTer52

## Abstract

**Summary:**

Brooke–Spiegler syndrome (BSS) is an autosomal dominant disease associated with the *CYLD* gene, which manifests itself as multiple benign skin tumors. We presented a young female patient with a previously undescribed heterozygous *de novo* mutation c.2501dupC (p.Val835SerfsTer52) of the *CYLD* gene. The nucleotide sequence variant was detected by clinical exome sequencing and validated by Sanger sequencing in her parents and sister. A histological examination of the elements identified multiple trichoepitheliomas. Radical removal of the largest formations of the facial skin was performed under local infiltration anesthesia with wound treatment with a CO_2_ laser in a pulsed-periodic mode. Nevertheless, new formations began to appear on the patient’s facial skin of the forehead, upper eyelids, nasolabial triangle, prone to growth and requiring removal. Domain modeling of the mutant protein proved its conformational difference from the wild type, as well as altered physicochemical properties.

**Conclusion:**

The new *CYLD* gene variant p.Val835SerfsTer52 causes the development of multiple familial trichoepitheliomas in BSS and confirms the hypothesis of the association of this gene variant with loss-of-function mutations. We have verified that removing multiple trichoepitheliomas simultaneously with a CO_2_ laser does not impede the pathological process. Therefore, hopes are placed on targeted therapy, which is currently not developed for this disease.

## 1 Introduction

Genodermatosis is an extensive clinically heterogeneous group of hereditary diseases characterized primarily by lesions of the skin and dermal appendages. Dermatoscopy is an important diagnostic tool for genodermatoses, particularly when the symptoms are not pronounced (1). Clinical manifestations of genodermatosis depend on the type and location of the mutation in a particular gene. The *CYLD* gene is a tumor suppressor and encodes an ubiquitin-specific protease which main purpose is to activate apoptosis (2). Germline mutations in the heterozygous state of *CYLD* gene are described in following cases of patients with Brooke–Spiegler syndrome (BSS): the classic clinical variant, multiple familial trichoepithelioma, familial cylindromatosis (3). It is believed that all clinical variants comprise one phenotypically heterogeneous genodermatosis with an autosomal dominant inheritance (4, 5).

All clinically significant mutations of the *CYLD* gene debut during puberty, the average onset age is 16, sex ratio (number of women to men) varies from 0.33 to 4.00 (6). Clinically, *CYLD* gene mutations manifest by multiple benign tumors of the skin and its appendages. Although it is possible for tumors to become malignant and develop into adenocarcinoma, cylindrocarcinoma, sarcomatoid carcinoma, squamous cell skin cancer, trichoblast carcinoma, which reduces the life expectancy of patients (7). There is a case of development of a membranous type basal cell adenoma of the salivary gland in BSS, which is described in the literature (8). An annual dermatological examination is recommended for individuals diagnosed with BSS, with follow-up frequency adjusted based on patient needs—ranging from every few months for those needing repeated surgeries to as needed for stable cases. Patients should report any changes in tumors, such as rapid growth or bleeding, and undergo additional assessments if necessary; annual salivary gland exams and lung imaging are advised for those over 40 with new breathlessness (7).

According to the VarSome database, 65 pathogenic and 8 likely pathogenic variants of the *CYLD* gene (the most common mutation type is frameshift), as well as 145 variants with unknown clinical significance (mostly missense mutations) are registered at the moment (date of access 29 August 2024) (9). Literature contains reports on 107 mutations that are associated with skin pathology (10). There is no correlation between skin manifestations and genomic position of the variant, however, 99% of mutations occur between exon 9 and 20 (NM_015247).

This study presents a previously undescribed *de novo* variant c.2501dupC (p.Val835SerfsTer52) of the *CYLD* gene in a young woman with multiple trichoepitheliomas. This variant was detected by clinical exome sequencing and validated by Sanger sequencing.

## 2 Case presentation

Patient N., Caucasian woman, born in 2001, consulted a dermatovenerologist on 09/08/2019 with complaints of skin rashes on their face (11).

First skin rashes appeared on the face at about the age of 11, in 2012. Patient visited a local dermatologist and was diagnosed with acne. Despite prescribed treatment (Azelaic acid, 20% cream, twice daily, morning and evening), rash did not regress. Patient consulted a cosmetologist, who conducted a course of TCA peels, after which the patient noted growth progression and increase in the number of rashes. On 05/03/2018, the patient saw another dermatologist who decided to perform a biopsy. The biopsy of the facial skin lesions conducted at the local hospital confirmed a diagnosis of syringoepithelioma, with the formation located in the dermis and having a solid structure composed of round, monomorphic cells, some of which are arranged in a palisading pattern, and concentric masses of keratohyalin identified in the center of some complexes. No further studies were carried out at the local hospital, lesion was regarded as a singular, biopsy samples were not preserved, blocks were not requested by the patient.

She was born full-term via vaginal delivery following an uncomplicated pregnancy and labor. She was the second child of healthy, non-consanguineous parents (28-year-old mother, 32-year-old father). She grew and developed according to age; at the moment she is a student. She has no history of allergies and denies the presence of skin diseases in relatives. Her past diseases: acute respiratory viral infections, chicken pox. No genetic testing had been previously performed on any member of the family, including the patient herself.

The patient’s condition is satisfactory. The physical examination revealed a height of 170 cm, a weight of 60 kg.

Abdominal and kidney ultrasound: no pathology. Pelvic, lymphatic vessel, thyroid, and salivary gland ultrasounds: no pathology. Echocardiography: no organic pathology. Electrocardiography: no arrhythmias detected. Stool examination: no pathology; no helminthic or parasitic infections. Complete blood count, biochemical blood test, and urinalysis: no pathology. Chest X-ray: no pathology.

**Figure 1:**
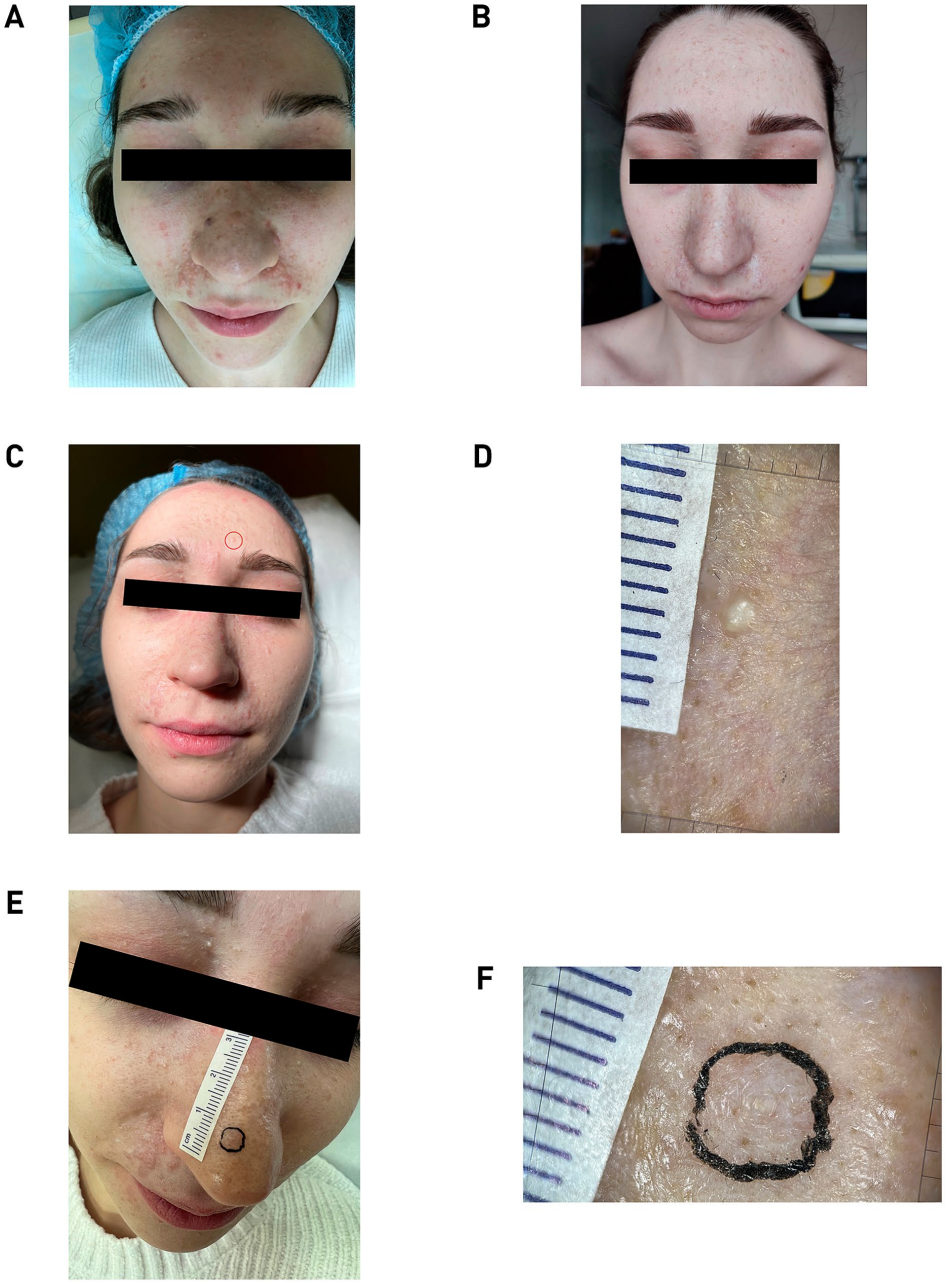
**(A)** Patient N., 19 years old, 2019. Pathological process is widespread on the skin. Photo was taken before the removal of skin lesions. **(B)** Two years post-treatment, showing normotrophic scars and new papules on the forehead, upper eyelids and cheeks. **(C)** Patient N., 22 years old, 2024. The skin formation on the forehead, enclosed in the red circle, was subsequently taken for histological examination. **(D)** Dermatoscopic image of forehead lesion showing pearly inclusions. **(E)** Image of skin formation on the nose marked for histology. **(F)** Dermatoscopic image of the nose bridge lesion with central white inclusions. All dermatoscopic images was captured using an optical device with 20× magnification (FotoFinder; Germany) coupled with a smartphone controlled by the mobile application Handyscope pro.

Pathological skin condition is widespread. Lesions are localized on the facial skin, relatively symmetrically. Rashes are mostly located in the nasolabial triangle spreading to the area below eyebrows (X-shaped) (Figure 1A). In total, there are more than 80 hemispherical dense dermal papules on the patient’s skin, from skin-color and light pink to brown, 1–10 mm in diameter. Surface of the papules is smooth, dilated blood-vessels are visible in larger ones, and some lesions have lobulation and milia-like inclusions. Skin of the body, upper and lower extremities is without rashes. Mucous membranes are clean.

Differential diagnosis was conducted with other hereditary syndromes associated with multiple skin appendage neoplasms: Carney syndrome, Clouston syndrome, Cowden syndrome, Bazex–Dupré–Christol syndrome, Gardner syndrome, Gorlin–Goltz syndrome, Birt–Hogg–Dubé syndrome, Muir–Torre syndrome, Schöpf–Schulz–Passarge syndrome, tuberous sclerosis, and TSC2/PKD1 contiguous gene syndrome. While the clinical and histological features were most indicative of BSS, there was no significant family history in patient N.

The patient’s computed Dermatology Life Quality Index (DLQI) score of 11 indicates that the disease had a very large effect on her life prior to surgical therapy. She specifically states that her skin issue caused her to experience great embarrassment and self-consciousness. Her skin problems also had a significant negative impact on her social and recreational activities, created considerable challenges in her relationships with close friends and partner, resulted in major sexual difficulties. The findings point to a psychosocial burden and emphasize the necessity of focused therapeutic interventions.

The radiation power was selected for each lesion and varied in the range from 1.5 to 3 W. Initially, several small neoplasms on the cheek skin were removed, after which the patient was under dynamic observation for 6 months to determine the risk/benefit ratio. A positive cosmetic outcome was achieved; the lesions did not recur, and their removal did not stimulate the growth of nearby tumors, which allowed the continuation of lesion removals using the chosen methodology. In the second stage, 6 months after the first removal, all large lesions located in the nasolabial triangle area were removed. The final, third stage of lesion removal was conducted with a 6-month interval after the second stage and included the removal of the lesions that were the most aesthetically discomforting for the patient. The quality of the lesion removals was assessed at 1 and 6 months post-procedure through visual inspection and dermatoscopy (Figure 1B).

By age 22, a macroscopic examination showed that the pathological skin condition was widespread, with eruptions appearing relatively symmetrically. The highest concentration of lesions was observed in the nasolabial triangle area (Figure 1C). Dermatoscopic analysis revealed a lobular structure with pearly inclusions in the forehead lesion, which was subsequently taken for histological examination (Figure 1D). Additional dermatoscopic imaging of a lesion on the nose bridge showed a non-structured volumetric formation with central white inclusions (Figures 1E,F).

Currently, patient N. is satisfied with the treatment outcomes (DLQI = 3) and continues to be under our dynamic observation. The patient’s skin condition no longer interferes with social activities, although she still feels a little amount of embarrassment and self-consciousness, along with minor anxieties when interacting with her partner. After the final course of therapy, the patient got married, suggesting further positive outcomes in her personal life.

## 3 Methods

### 3.1 Histology

Removed fragments of the skin lesions were placed in a 10% solution of neutral formalin for fixation in 24 h. After isopropanol standard histological processing in vacuum tissue processor Tissue-Tek VIP 6 (Sacura, Japan), the tissue paraffin blocks were made. Sections were made using rotary microtome CUT 4062 (SLEE Medical GMBH, Germany), with a thickness of 3 microns. Staining with hematoxylin and eosin according to the standard procedure was made with tissue automated stainer Tissue-Tek Prisma (Sacura, Japan).

### 3.2 Molecular study

DNA was isolated from peripheral venous blood using standard techniques with QIAamp DNA Mini Kit (Qiagen, Hilden, Germany). The extracted DNA was measured with a Qubit dsDNA BR Assay system (Life Technologies, Carlsbad, CA, United States), and its quality was evaluated by running a 1% agarose gel electrophoresis. Adapters were added to DNA fragments obtained after restriction enzyme treatment, after which DNA libraries were prepared using a QIAseq FX DNA Library Combinatorial Dual-Index Kit (Qiagen). Enrichment was performed using SureSelect XT2 kit (Agilent Technologies, Santa Clara, CA, United States). DNA sequences were analyzed with HiSeq 2500 (Illumina, San Diego, CA, United States). FastQC version 0.11.9 was utilized to assess the quality of the paired fastq files obtained (12). After sequencing, Cutadapt (13) was used to trim 3′-end nucleotides that had a quality score below 10. Reads were aligned to the GRCh38.p13 human reference genome using bwa-mem2 v2.2.1 (14). The width of the clinical exome coverage was 98.26% and the average depth was 98.26×. The. bam file was employed for variant calling following the Genome Analysis Toolkit (GATK) best practices (15). Variants were classified according to the guidelines of the American College of Medical Genetics and Genomics (ACMG) (16).

### 3.3 *In silico* analysis

Analysis of the evolutionary conservation of the CYLD protein was carried out using the ConSurf server (17). The HMMER homologue search algorithm with single iteration and an E-value cut of 0.0001 was used, UNIREF90 was chosen as the protein database. Wild-type protein (USP domain, 2VHF.pdb) was visualized with ChimeraX, and the SWISS-MODEL workspace online resource (18) was used to predict the structure of the mutant protein and build Ramachandran maps. Hydrophobicity, refractoriness, recognition factors and percentage of hydrophilic amino acid residues were calculated using the ProtScale server (19). Biological pathway enrichment evaluation was performed using ShinyGO v0.75 (20). Date of access of all the online resources listed above: 02/22/2023.

## 4 Results

**Figure 2:**
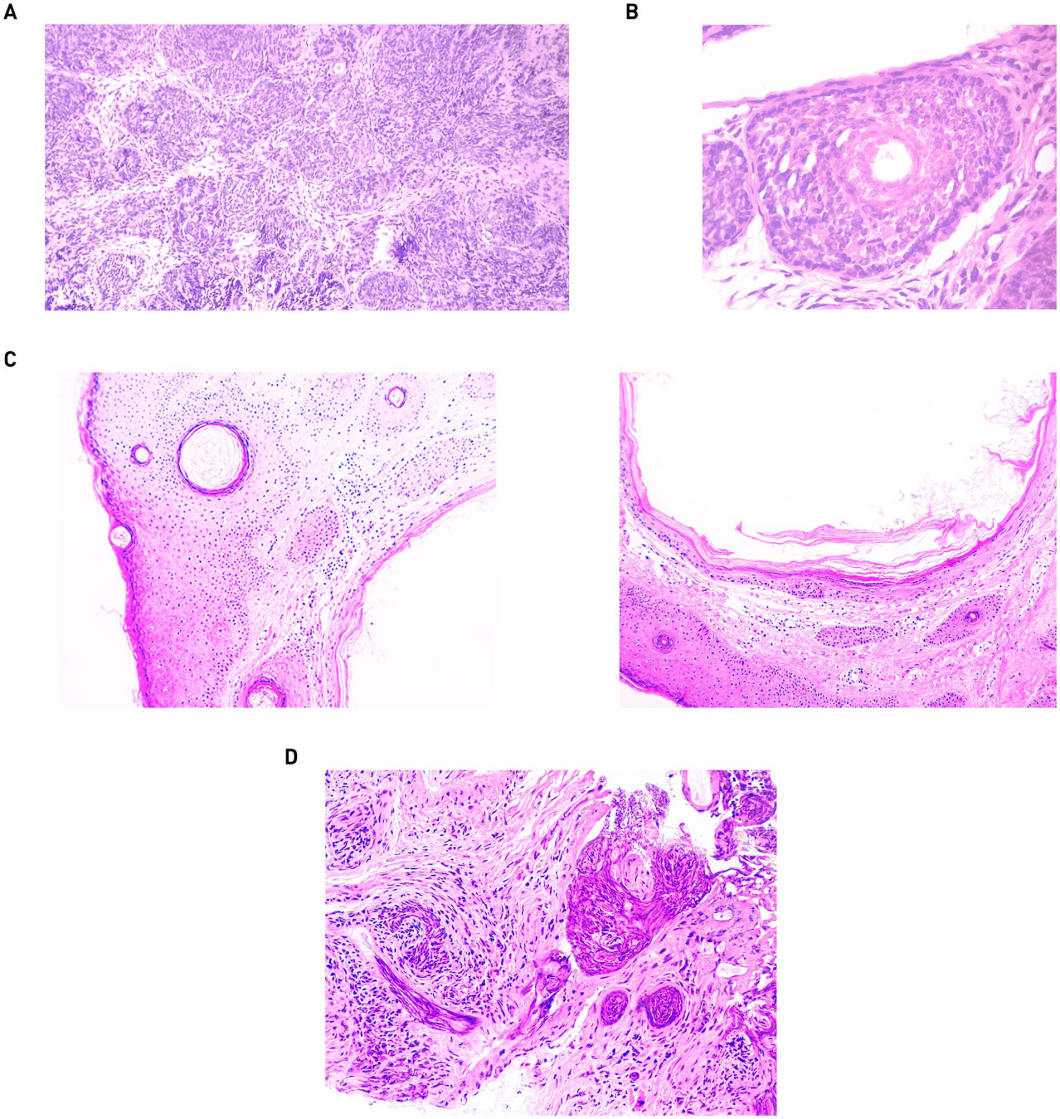
Skin biopsies from 2022: **(A)** magnification 100. **(B)** Magnification 400. Skin biopsies from 2024: **(C)** skin fragment from the forehead with keratin cysts. Magnification: 100. **(D)** Skin fragment with elements of recurrent trichoepithelioma. Magnification: 200.

Skin samples of the dermis, taken 2 years after the first treatment with CO_2_ laser, revealed trichius-like histological lesions that merge with each other, forming palisade-like structures from basaloid cells (Figure 2A), as well as forming follicle-like structures with the production of a scanty hair matrix in the center (Figure 2B). In the histological examination of the skin fragment from Figure 1D, acanthosis and moderate hyperkeratosis of the multilayered squamous epithelium with the formation of multiple keratin cysts containing laminated keratin masses in the lumen were identified (Figure 2C). In the skin sample from the nose bridge, multiple epithelial cords of multilayered squamous epithelium with deformative changes were identified, most consistent with recurrent trichoepithelioma (Figure 2D).

A previously undescribed mutation c.2501dupC (p.Val835SerfsTer52) was detected in the heterozygous state (frameshift insertion in exon 17, transcript NM_001042355.2), which was annotated as likely pathogenic in the VarSome database. This variant causes the cessation of protein synthesis after 52 codons from the modified one (PVS1 criterion). This variant was absent in public databases (gnomAD, RUSeq, dbSNP, ClinVar) (PM2 criterion).

**Figure 3:**
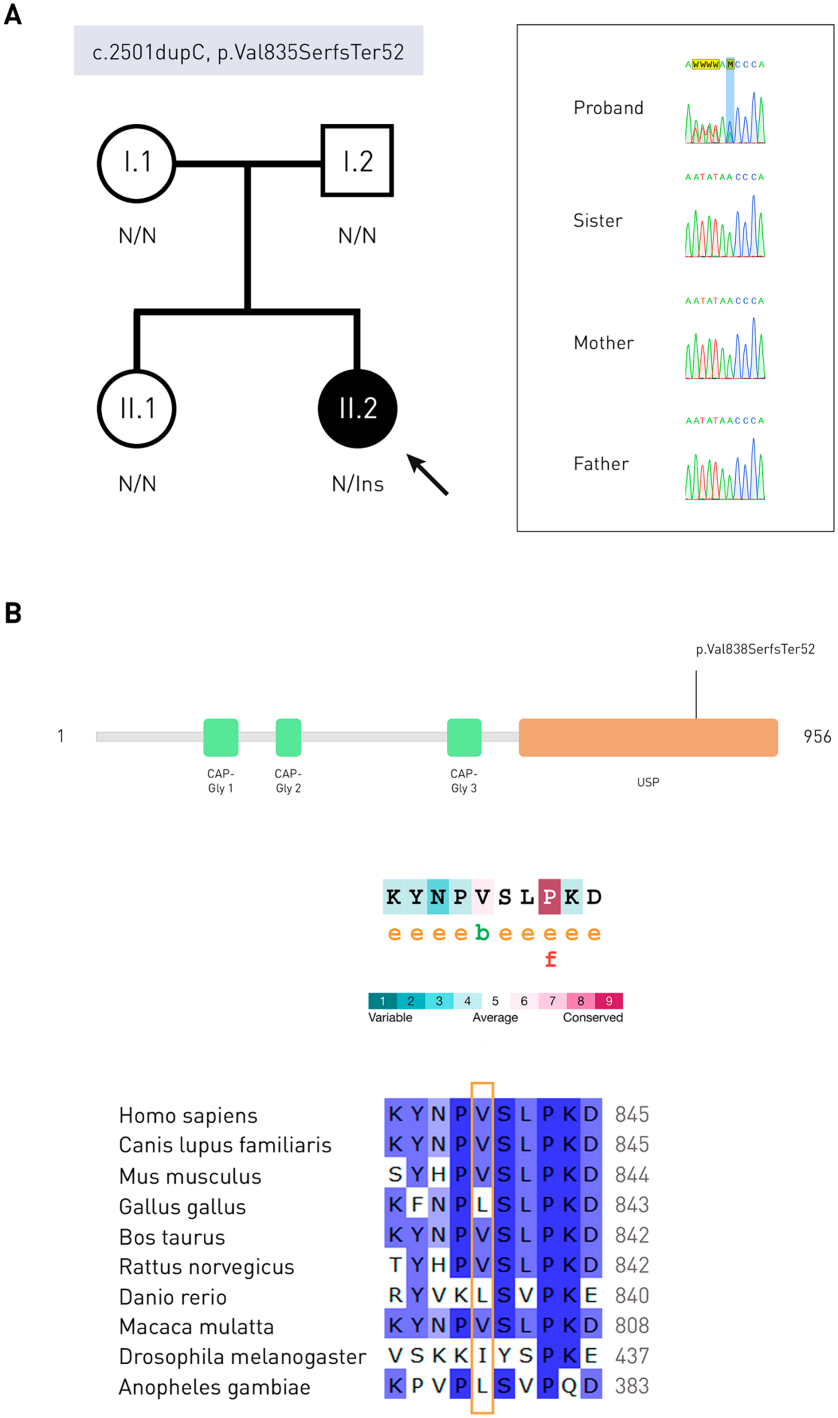
Molecular genetic studies and *in silico* analysis. **(A)** Pedigree of the patient and results of Sanger sequencing in the form of electrophoregrams. The proband is indicated by a solid circle. N is the normal allele of the gene. Ins, insertion. **(B)** Schematic representation of protein domains (Q9NQC7, UniProtKB) and evolutionary conservatism of the CYLD amino acid sequence: the valine residue is moderately conserved. e—residue is hydrophilic according to the NACSES algorithm. b—residue forming hydrophobic cores, according to the NACSES algorithm. f is the residue predicted to be functional (with high conservatism and hydrophilicity). Followers of various CYLD protein orthologs, nucleotide positions are colored according to the frequency of occurrence among all species: ≤40%—white, 41–60%—light-blue, 61–80%—blue, 81–100% dark blue (UGENE).

To validate the variant, Sanger sequencing of exon 17 of the *CYLD* gene (NM_001042355.2) of the patient’s parents and sister was performed (Figure 3A). Due to the fact that the variant was not found in parents, it was recognized as *de novo*.

Change of the amino acid residue in protein occurred in the USP domain (ubiquitin-specific peptidase). Evolutionary conservatism analysis showed that this region containing amino acid residues 834–843 is variable, with a mean ConSurf of 4.9 (Figure 3B). In a multiple sequence alignment compiled with ConSurf, valine 835 occurred in 96.19% of the sequences (101/105), and could be replaced by neutral leucine, isoleucine, methionine (non-polar amino acids), and serine (polar amino acid). Serine in this position is found in representatives of the Cyprinoidei suborder. Out of 1838 homologues of the CYLD protein, 226 passed the identity threshold, after which redundant homologues with a threshold of 95% were removed using the Cluster Database at High Identity with Tolerance (CD-HIT). The average number of substitutions between any two sequences the alignment was 0.606 (0.007–1.828). As a result of the frameshift, reference amino acid sequence NPVSLPKDLPDWDWRHGCIPCQNMELFAVLCIETSHYVAFVKYGKDDSAWLFF changed to NPSVTSQRLTRLGLETRLHPLPEYGVICCSLHRNKPLCCFCEVWEGRFCLALL. Only leucine 844, proline 855, and phenylalanine 875 matched in sequence alignment (no gaps). The average ConSurf value of the native protein region 836–888 is 6.5, which indicates its tendency to conservatism. Analysis of the mutant protein evolutionary conservatism was also carried out. One hundred and eighty-five out of the 1,816 found homologues passed the identity threshold, and 93 were CD-HIT unique. The average pairwise distance of aligned sequences was 0.488 (0.022–1.571). After the mutation site, the most similarities were observed with a protein from the Polypteridae family.

The sequence, consisting only of altered amino acid residues, was more successfully aligned with A0A8S0ZK26 (hydroxyproline-rich glycoprotein, *Arctia plantaginis* sp.) (identity at positions 4, 22, and 48), but the ConSurf analysis failed due to the lack of unique homologues.

**Figure 4:**
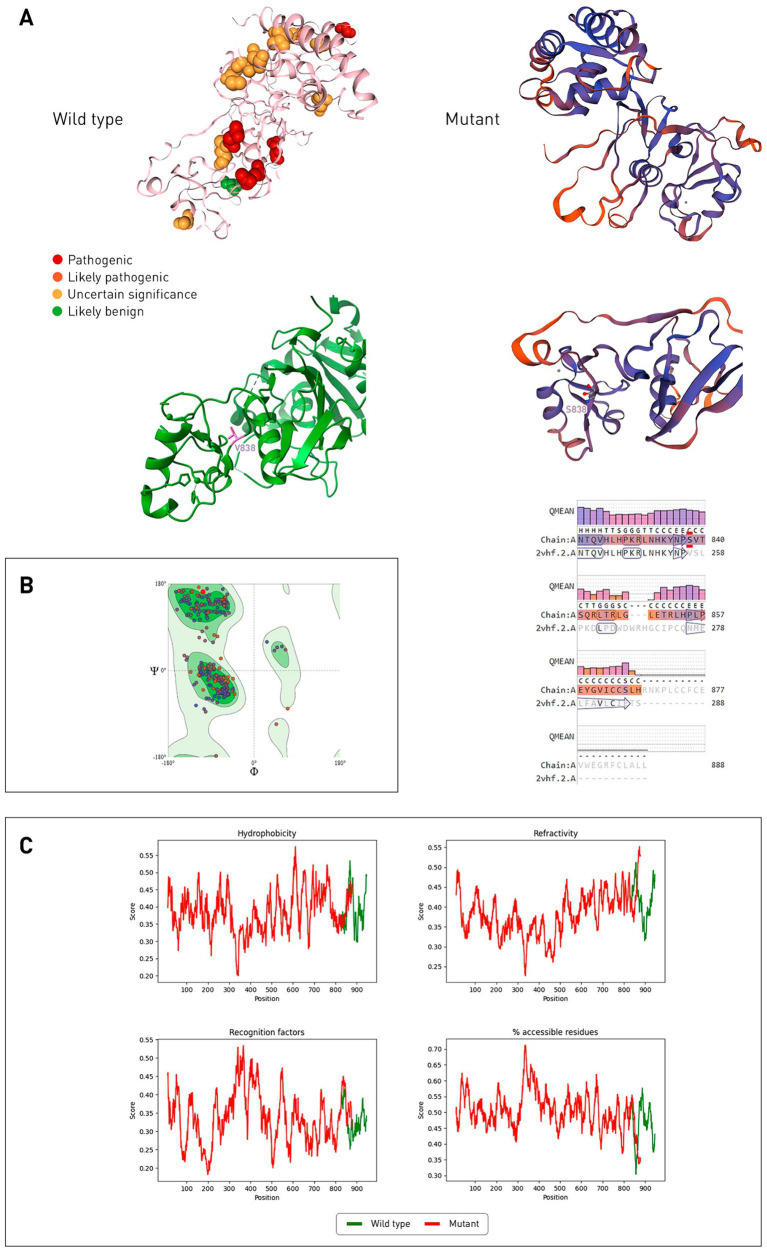
Comparison of wild-type and mutant CYLD protein structures (USP domain). **(A)** Gene variants located corresponding to the affected amino acid residues on the wild-type protein CYLD (2VHF.pdb), stained according to the ACMG classification. The mutant CYLD protein is shown on the right, its structure predicted by SWISS-MODEL, 2vhf.2.A was used as a template. Alignment of the model with a template with a QMEAN quality score for each position is presented below. Color reflects the degree of confidence in the prediction (low degree—red, high degree—dark blue). **(B)** Ramachandran general map of the CYLD mutant protein. Auspicious regions are indicated in green, light green—permissible regions, prohibited regions are indicated in white. **(C)** Hydrophobicity, refractoriness, recognition factors, and the percentage of hydrophilic amino acid residues of proteins of wild-type and mutant CYLD proteins were predicted by ProtScale.

The catalytic domain of CYLD is USP, it contains the BL1 (β8/β9) and BL2 (β10/β11) ubiquitin-binding loops, with the first amino acid residue of the BL2 loop being asparagine 858 (21). According to the VarSome online resource, the pathogenic variant closest to the mutation site is at position 896 (glycine) (Figure 4A). Using SWISS-MODEL, models of the USP domain of the mutant CYLD protein were built, among them the best one was selected in terms of QMEANDisCo Global, which was equal to 0.69 ± 0.05, and the sequence identity with the 2vhf.2.A sample was 90.49%. Ramachandran maps (Figure 4B) revealed that in the mutant protein model, 93.26% of the amino acid residues are in favorable regions. Outliers are GLU585, THR676, PRO855 and LEU866 (1.42%).

According to ProtScale, the hydrophobicity of the mutant CYLD protein decreases after the mutation site (Figure 4C) and the refractoriness increases. The mutant protein has a higher value of recognition factors (average stabilization energy for an amino acid) and the percentage of hydrophilic amino acid residues.

## 5 Discussion

Despite the fact that the main clinical manifestations of *CYLD* gene mutations are associated with the development of multiple adnexal skin lesions, there are cases of patients with frontotemporal dementia and/or amyotrophic lateral sclerosis type 81 (22) and with Alzheimer’s disease (23). It is assumed that the development of these neurological diseases is due to impaired fusion of autophagosomes with lysosomes (inhibition of the NF-kB signaling pathway is enhanced), but the mutant *TBK1*, *OPTN*, and *SQSTM1* genes also contribute to the pathogenesis. Mutations of the *CYLD* gene of the gain-of-function type occur in neurological patients, loss-of-function mutations in patients with skin pathology; the former are missense in type, and the latter are frame-shift (24). Mutation in patient N. corresponds to the proposed association with the phenotype.

Relatives with the same *CYLD* mutation show clinical heterogeneity (25). Since the patient N. variant was *de novo*, it is not possible to assess the penetrance and variability of the disease. Literature describes variants that modify the skin pathology phenotype: rs1053023 (*STAT3*), rs1131877 (*TRAF3*), and rs202122812 (*NBR1*) (26). *In vitro* analysis demonstrates that prognosis is directly correlated with the activity of the NF-κB signaling pathway (27).

*Cyld*^−/−^ knockout mice had inflammatory bowel disease, were more susceptible to induced mutagenesis, and also developed skin lesions (28). A decrease in the number of peripheral blood T cells (CD4^+^ and CD8^+^) has been reported, presumably caused by impaired induction of anti-CD3/CD28 antibodies in double positive thymocytes. Interestingly, in people with inflammatory bowel disease, *CYLD* expression was reduced (29). We presented an analysis of the evolutionary conservation of the protein, which confirmed the high amino acid sequence similarity between human and mouse CYLD. In multiple alignments, the CYLD mutant had almost no matching positions after the altered amino acid residue, from 843 position this figure was less than 10% of all sequences, according to ConSurf. Domain modeling of the mutant protein proved its conformational difference from the wild type, as well as altered physicochemical properties. The mutant protein loses two amino acid residues, one of which performs a catalytic function (aspartate 889), and the other is responsible for contact with ubiquitin (tyrosine 943) (21). Also, histidine 871 (catalytic function) is replaced by neutral proline, and tyrosine 872 (binding to ubiquitin) is replaced by leucine.

## 6 Conclusion

The discovered variant c.2501dupC (p.Val835SerfsTer52) of the *CYLD* gene is associated with a structural change in the protein, causes the loss of its function and causes the appearance of multiple trichoepitheliomas in a young woman. We have confirmed that the simultaneous removal of numerous trichoepitheliomas with a CO_2_ laser does not slow the pathological process, as new growths consistently appear each year. Additionally, over time, patients with BSS may develop neoplasms of different histological types.

## Data Availability

The original contributions presented in the study are included in the article/supplementary material, further inquiries can be directed to the corresponding author.
